# Primary undifferentiated spindle cell sarcoma obstructing the right ventricular outflow tract

**DOI:** 10.1186/s13019-020-01129-8

**Published:** 2020-05-04

**Authors:** Jintae Kwon, Kyewon Kwon

**Affiliations:** 1 Department of Thoracic and Cardiovascular Surgery, Bundang Jesaeng Hospital, Zip code: 13590, 20, Seohyeon-ro 180beon-gil, Bundang-gu, Seongnam-si, Gyeonggi-do Republic of Korea; 2 Department of Pathology, Bundang Jesaeng Hospital, Seongnam-si, Gyeonggi-do Republic of Korea

**Keywords:** Sarcoma, Right ventricular outflow tract, Spindle cell

## Abstract

**Background:**

Primary undifferentiated spindle cell sarcoma in the right ventricle is an extremely rare tumor. Radical surgical excision is the optimal treatment for long-term survival due to poor response to chemotherapy or radiotherapy at an advanced stage.

**Case presentation:**

A 42-year-old man with no previous medical history presented with mild dyspnea on exertion and abdominal distension that lasted a week. Computed tomography (CT) revealed a huge homogeneous mass completely obstructing the right ventricle and extending into the pulmonary trunk. However, he suddenly collapsed the next day while on his way to an echocardiography. An extracorporeal membrane oxygenation (ECMO) device was inserted percutaneously and ECMO support was urgently initiated. Based on consideration of right ventricular outflow tract (RVOT) obstruction in the initial CT scan, we decided to remove the mass from the right ventricle immediately. The main mass was resected to relieve the RVOT obstruction, and after the operation, the ECMO was removed from the operation room. However, the patient failed to regain consciousness and electroencephalography (EEG) and subsequent magnetic resonance imaging (MRI) indicated severe hypoxic brain damage. We assume CPR was unsuccessful because the mass completely blocked the RVOT. Pathology revealed the mass was an undifferentiated spindle cell sarcoma.

**Conclusions:**

We present the case of a 42-year-old male with cardiac arrest due to right ventricular outflow tract obstruction by a tumor of the right ventricle. Surgical resection was performed and in histopathology it was proved to be an undifferentiated spindle cell sarcoma.

## Background

Although several atrial spindle cell sarcomas have reported, primary undifferentiated spindle cell sarcoma in the heart is a very rare tumor [1, 2]. Radical surgical excision is the mainstay of treatment, but despite surgical resection, its prognosis is poor due to delayed diagnosis, early metastasis, and few available therapeutic options.

## Case presentation

**Fig. 1:**
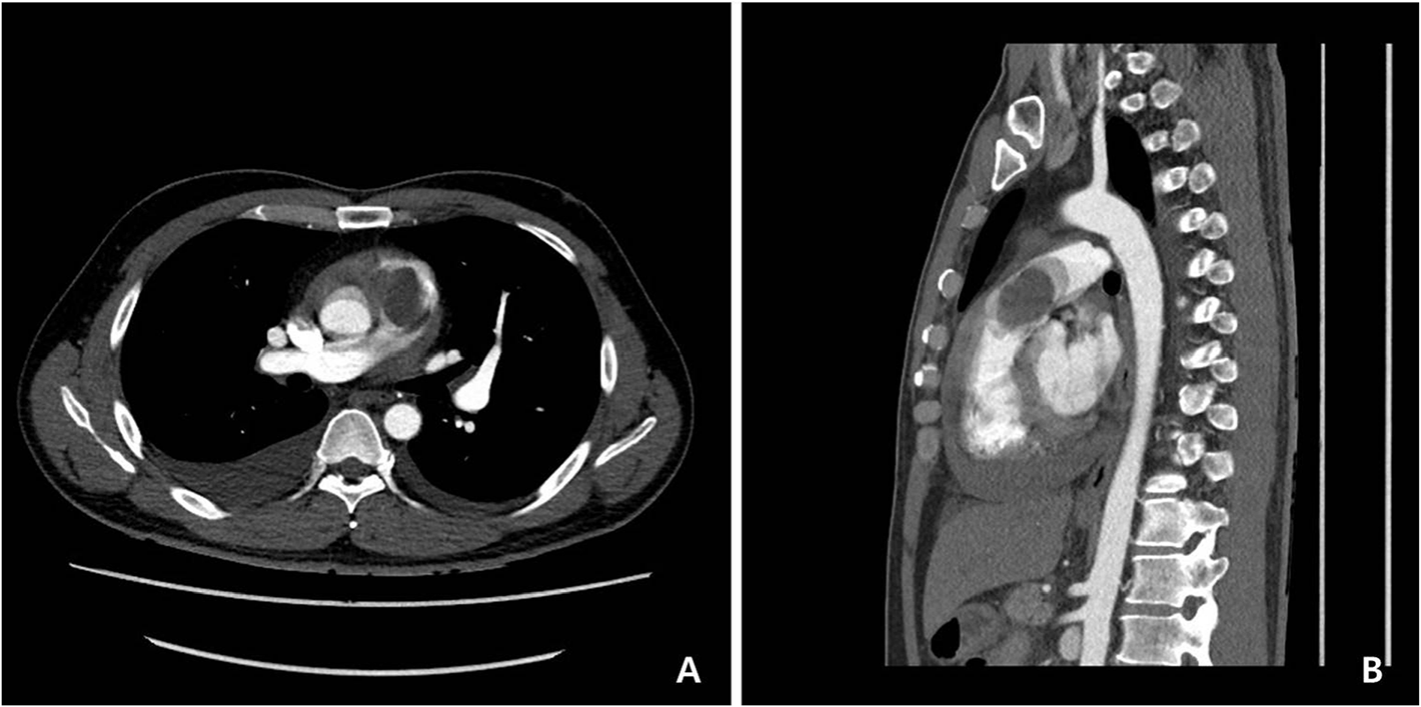
CT axial (**a**) and sagittal (**b**) images showing a mass completely obstructing the right ventricular outflow tract

**Fig. 2:**
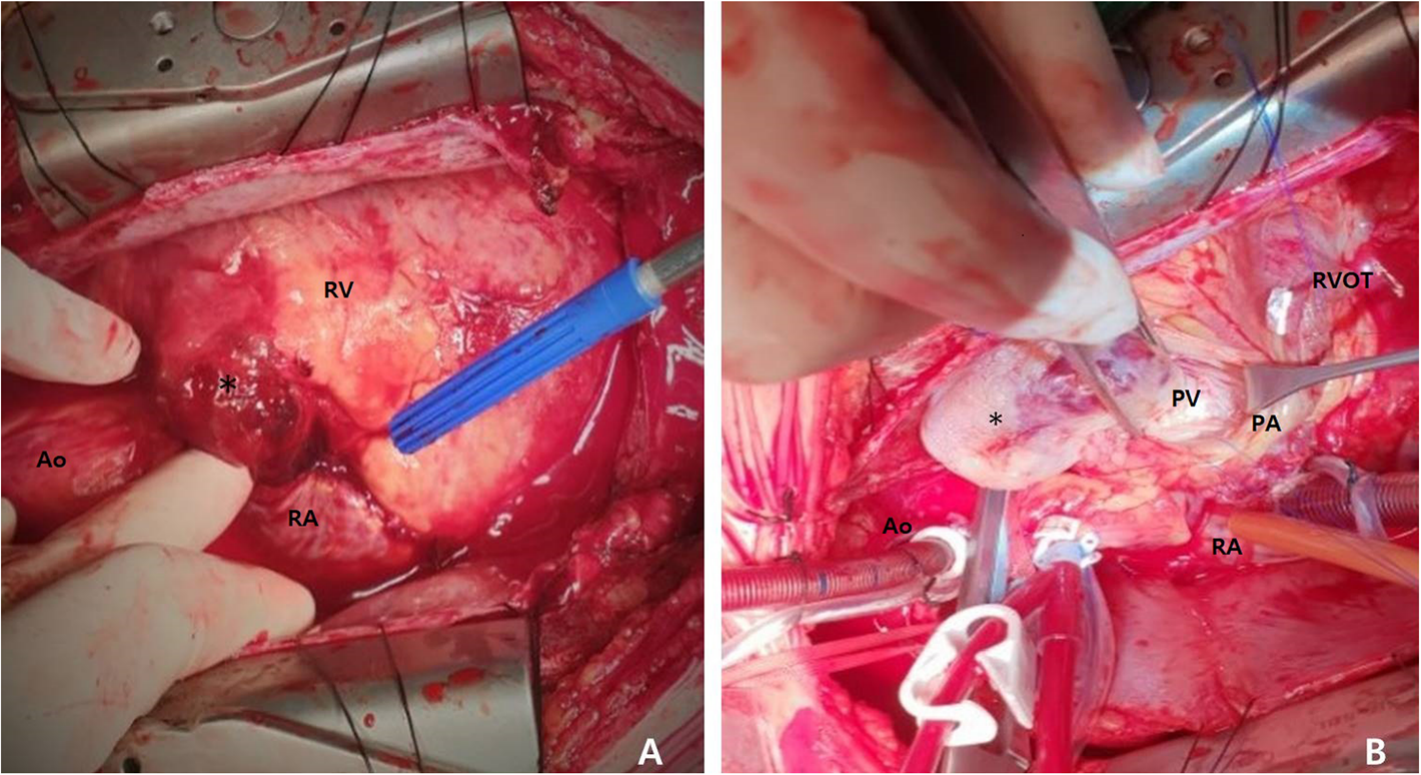
Intraoperative photographs showing involvement (asterisk) of epicardium of the right ventricle (**a**) and the tumor (asterisk) located in the right ventricular outflow tract (**b**) Ao; ascending aorta, RA; right atrium, RV; right ventricle, RVOT; right ventricular outflow tract, PA; pulmonary artery, PV; pulmonic valve.

**Fig. 3:**
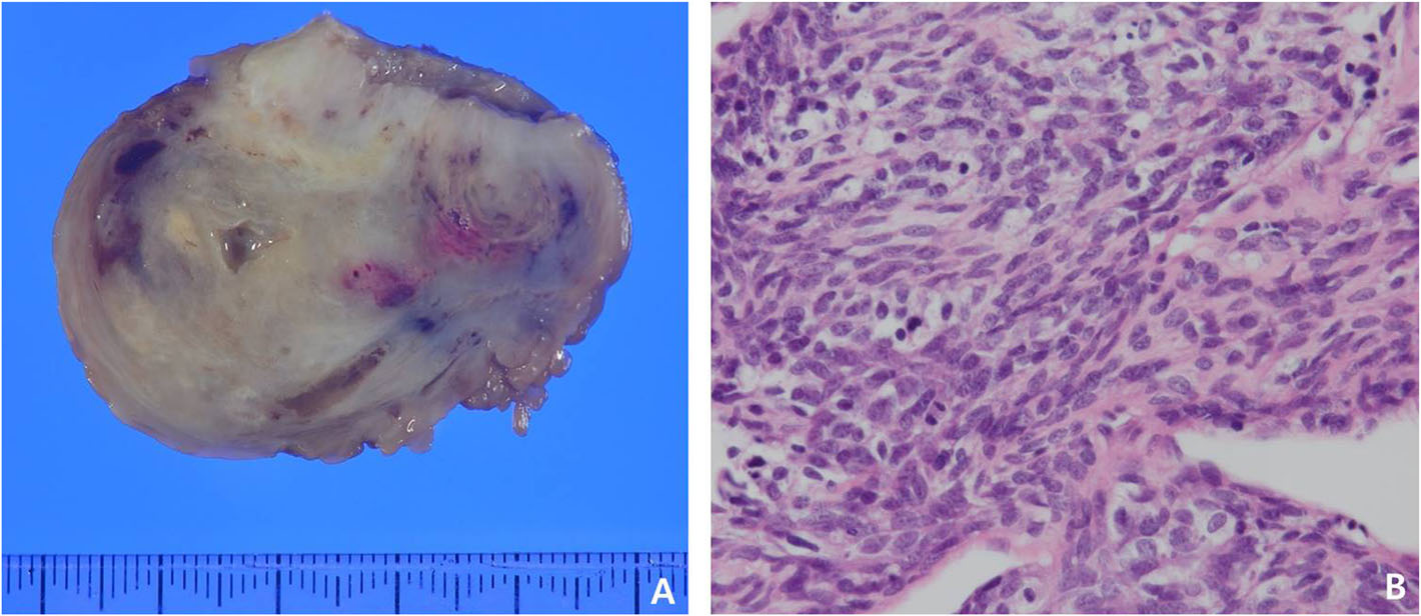
Gross pathology of cardiac spindle cell sarcoma (**a**) and histopathology of cardiac spindle cell sarcoma (Hematoxylin and eosin stain, × 400) (**b**)

A 42-year-old man with no previous medical history presented with mild dyspnea on exertion and abdominal distension that lasted for a week. Computed tomography (CT) revealed a huge homogeneous mass completely obstructing the right ventricle and extending into the pulmonary trunk (Fig. 1a, b). CT findings showed little evidence of blood clots and moderate amounts of pericardial effusion (Fig. 1b). The patient was admitted via the emergency room for further evaluation and scheduled for echocardiography the next day. However, he suddenly collapsed the next day while on his way to an echocardiography. Cardiopulmonary resuscitation (CPR) was performed immediately by medical staffs, but heart rhythm did not recover. Accordingly, an extracorporeal membrane oxygenation (ECMO) device was inserted percutaneously via the left femoral artery and right femoral vein and ECMO support was urgently initiated. Subsequently, a pericardial window was created at bedside, and about 350 cc of dark blood colored effusion was drained. Soon afterward, blood pressure stabilized but consciousness was not confirmed. But his light reflexes remained intact. Based on consideration of right ventricular outflow tract (RVOT) obstruction in the initial CT scan, we decided to remove the mass from the right ventricle immediately. Surgery was performed using a median-sternotomy approach. Initially, an arterial cannula was placed in the distal aspect of the ascending aorta, and the superior and inferior venae cavae were cannulated to establish cardiopulmonary bypass (CPB). After aortic cross-clamping, the pulmonary trunk and RVOT were incised. By intra-operative gross visualization, the tumor was located in the RVOT and protruded from endocardium of the right ventricle out of epicardium of the right ventricle (Fig. 2a) and also extended toward and was attached to the right leaflet of the pulmonary valve (Fig. 2b). The tumor was fragile and considered highly likely to be malignant and impossible to completely control, and thus, we planned chemotherapy after surgery. The main mass was resected to relieve the RVOT obstruction, and after the operation, the ECMO was removed from the operation room. However, the patient failed to regain consciousness and electroencephalography (EEG) and subsequent magnetic resonance imaging (MRI) indicated severe hypoxic brain damage. We assume CPR was unsuccessful because the mass completely blocked the RVOT. Pathology revealed the mass was an undifferentiated spindle cell sarcoma (Fig. 3a, b).

## Discussion and conclusions

Twenty-five percent of primary cardiac tumors are malignant, and 95% of these are due to sarcoma [3]. Angiosarcoma is the most common type and accounts for around 40% of sarcomas, others include undifferentiated sarcoma (24%), malignant fibrous histiocytoma (14–24%), leiomyosarcoma (8%), osteosarcoma (6%), and least of all, spindle cell sarcoma [1, 3, 4]. Cardiac sarcoma can present with sudden onset, intermittent or positional symptoms of chest discomfort, dyspnea, orthopnea, cough, or syncope, heart failure, pericardial effusion and tamponade, arrhythmias, valvular abnormalities, or obstructive symptoms depending on heart chamber involved. Diagnosis is usually established using non-invasive imaging modalities such as transthoracic or transesophageal echocardiography. CT and MRI are useful for complete assessment of tumors occupying the entire cardiac chamber, tumors infiltrating myocardium or pericardium, or tumors with adjacent great vessel involvement [5]. At time of diagnosis, up to 80% of spindle cell sarcomas exhibit evidence of metastasis [2]. Tumor emboli are common and cause distant metastases involving bone, peritoneum, liver, and mesenteric lymph nodes [2]. Furthermore, spindle cell sarcoma is a highly aggressive and can rapidly metastasize. In conclusion, we report a rare case of spindle cell sarcoma obstructing the right ventricular outflow tract. Although treatment of this neoplasm of heart remains challenging due to its rarity and aggressive nature, radical surgical excision remains the mainstay treatment.

## Data Availability

Not applicable.

## Abbreviations

CT: Computed tomography
CPR: Cardiopulmonary resuscitation
ECMO: extracorporeal membrane oxygenation
RVOT: right ventricular outflow tract
EEG: electroencephalography
MRI: magnetic resonance imaging
CPB: cardiopulmonary bypass
